# The Importance of Treating Subclinical Hypothyroidism in Patients With Immune Thrombocytopenia: A Case Report

**DOI:** 10.7759/cureus.59813

**Published:** 2024-05-07

**Authors:** Nishtha Manuja, Varun Daiya, Suprit Malali, Ajinkya Kadu, Sunil Kumar, Sourya Acharya

**Affiliations:** 1 Department of Medicine, Jawaharlal Nehru Medical College, Datta Meghe Institute of Higher Education & Research, Wardha, IND

**Keywords:** sub clinical hypothyroidism, auto-antibody, eltrombopag, immune thrombocytopenia (itp), hashimoto's hypothyroidism

## Abstract

The leading cause of isolated thrombocytopenia in asymptomatic individuals is immune thrombocytopenia (ITP). It is an autoimmune disease characterized by decreased platelet counts caused by the immune system's destruction of platelets.

Sometimes, autoimmune thyroid diseases and ITP can coexist, which could cause an aggravated immune system response. When thyroid autoimmune diseases are present, treating ITP may become challenging. Treatment of the underlying thyroid disease in such individuals results in a significant improvement in platelet count, along with remission of the disease. It enhances the response to traditional ITP therapy. In this case report, we present a case of a 40-year-old female who was treated for ITP along with hypothyroidism, resulting in a considerable improvement in platelet count and a remission of the condition.

## Introduction

Immune thrombocytopenia (ITP) is an autoimmune disease characterized by decreased platelet counts which is caused by the immune system's destruction of platelets [1]. Primary ITP refers to the majority of idiopathic cases of ITP that have no underlying etiology. Conversely, secondary ITP is characterized by an underlying cause or disorder, such as drug-induced or systemic illness-induced (e.g. HIV, systemic lupus erythematosus, hepatitis C virus (HCV), medications, and cancers) [2]. Before confirming a diagnosis of ITP, it is crucial to consider and eliminate other apparent causes of thrombocytopenia as different etiologies of thrombocytopenia have distinct therapy strategies [3]. ITP can cause anything from being asymptomatic to potentially fatal spontaneous bleeding. There are a small number of case reports that linked Hashimoto's thyroiditis and Graves' disease to ITP [4]. As per recent research, treating thyroid autoimmune illnesses has been demonstrated to enhance the overall prognosis and clinical course of ITP [4].

## Case presentation

A 40-year-old female came with complaints of hyperpigmented patches (bluish-black) over the medial aspect of her left arm and groin region for seven days. The patient also gave a history of generalized weakness and fatigue for two months, along with weight gain for the same duration. For six months, the patient experienced menorrhagia, which was being managed conservatively and wasn’t evaluated for the cause. There was no history of fever, joint pain, or similar complaints of such patches in the past. Additionally, there was no history of diabetes mellitus, hypertension, tuberculosis, or bronchial asthma in the past.

**Figure 1:**
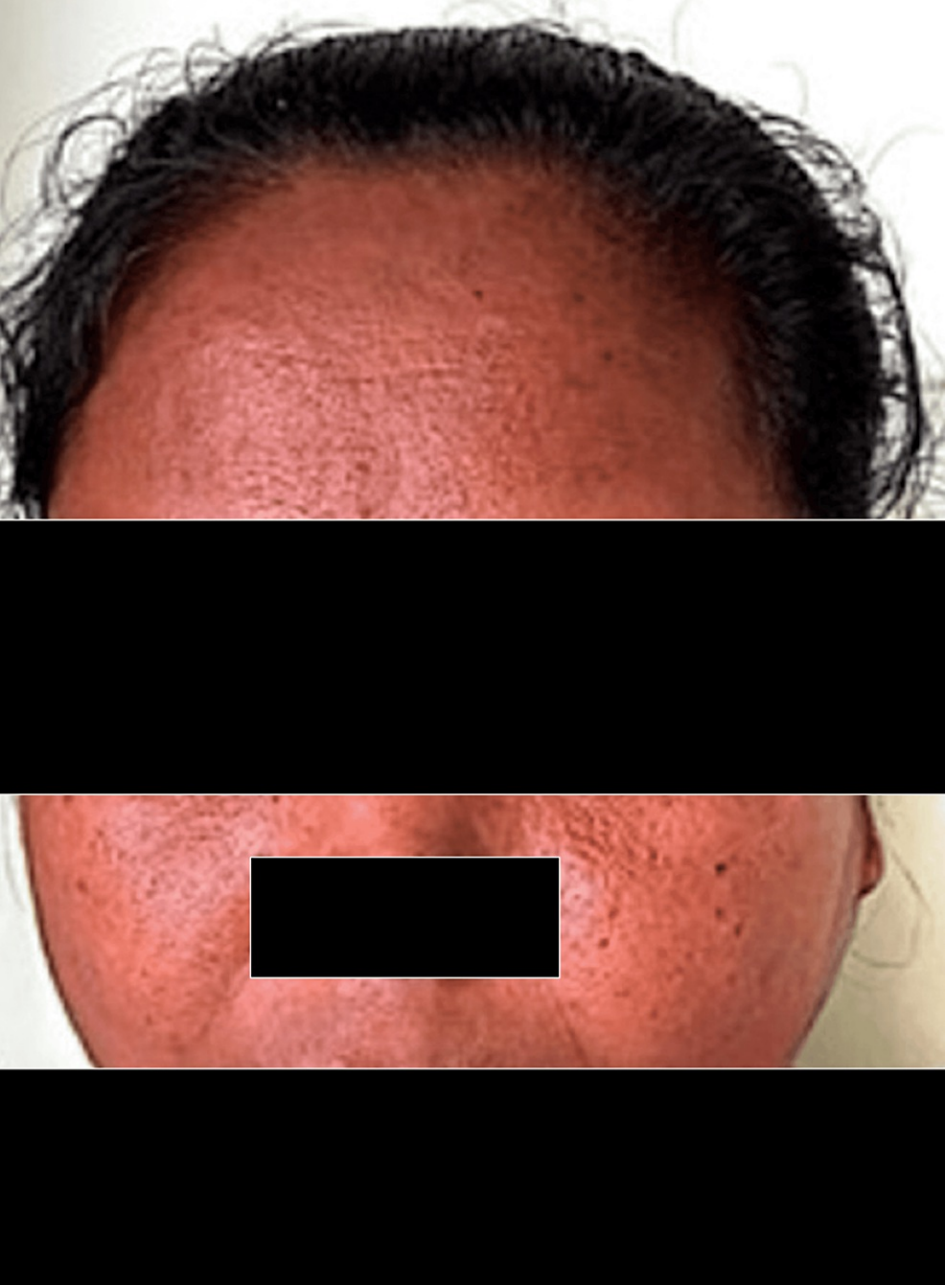
This figure shows a puffy face with a coarse skin texture of the patient.

On general examination, the patient had a puffy face and coarse skin texture, along with dry and brittle skin and hair (Figure 1).

**Figure 2:**
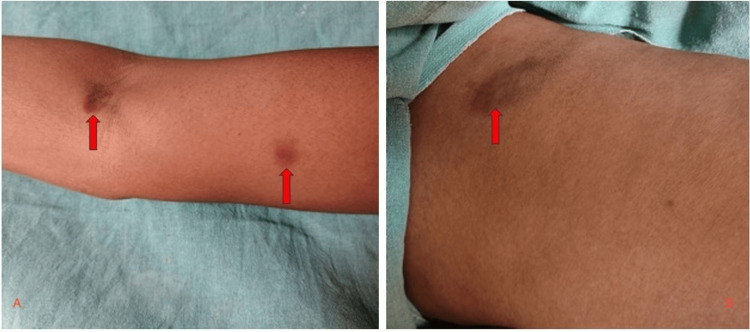
Figures A and B show the echymotic patches over the arm and thigh, respectively (highlighted by red arrow).

On local examination of the arm and thigh, a patch was observed on the medial aspect of the left arm, roughly 2x1 cm in size, and on the medial aspect of the left thigh, roughly 2x2 cm in size (Figure 2). 

The laboratory reports revealed decreased platelet counts of 5,000 per cu mm with normal hemoglobin and leukocyte counts. The peripheral smear showed normal morphology platelets with reduced counts as depicted in Table 1. The blood investigation also showed elevated thyroid stimulating hormone (TSH) with normal free thyroxine (T4) and triiodothyronine (T3) levels.

**Table 1:** Laboratory parameters of the patient.

Investigation	Normal range	Patient values
Hemoglobin	12-15 gm%	13.6 gm%
Platelet	150,000-410,000/cu mm	5,000/cu mm
Total leucocyte count	4,000-10,000/cu mm	8,900/cu mm
Serum urea	15-36 mg/dL	25 mg/dL
Serum creatinine	0.52-1.04 mg/dL	0.6 mg/dL
Serum sodium	137-145 mmol/L	140 mmol/L
Serum potassium	3.5-4.1 mmol/L	3.9 mmol/L
Serum alanine transaminase	Less than 35 U/L	12 U/L
Serum aspartate aminotransferase	14-36 U/L	24 U/L
Serum alkaline phosphatase	38-126 U/L	135 U/L
Serum total bilirubin	0.2-1.3 mg/dL	0.8 mg/dL
Random blood sugar	70-150 mg/dL	117 mg/dL
International normalized ratio		1.0
Thyroid-stimulating hormone	0.46-4.6 u/mL	8.90 u/mL
Serum free triiodothyronine	2.7-5.27 pg/mL	4.93 pg/mL
Serum free thyroxine	0.78-2.19 ng/mL	1.82 ng/mL
Serum prolactin	3-17.9 ng/mL	7.5 ng/mL
Peripheral smear	-	Normocytic normochromic, platelets reduced on smear with normal morphology
Serum thyroid peroxidase antibodies	Less than 30 IU/mL	991.3 IU/mL
Serum thyroid receptor antibodies	Less than 2.58 IU/L	1.16 IU/L

**Figure 3:**
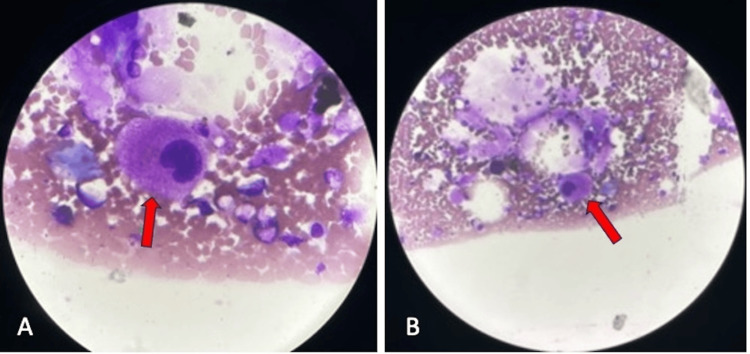
Figures A and B show a bone marrow biopsy with abnormal giant megakaryocytes at 100× and 20×, respectively (highlighted by red arrow). The image shows an electron microscope picture of bone marrow biopsy in oil emulsion and a normal microscope, respectively

Bone marrow aspirate and biopsy were done to rule out other causes of ITP and were suggestive of the increased number of abnormal giant megakaryocytes, hyperlobulated, hypo granular, and premature in morphology, which favored the diagnosis of immune thrombocytopenia (Figure 3).

The patient was administered intravenous methylprednisolone, 20 mg/kg/day, for four days, followed by oral prednisolone on a maintenance dose of 2 mg/kg/day for two weeks, gradually tapering over the next two weeks, along with levothyroxine supplementation of 75 mcg per day, and platelet counts showed a significant rise from 5,000/cu mm to 114,000/cu mm on her follow-up visit at four weeks.

## Discussion

The pathophysiology of ITP, which is a destructive platelet disorder, is primarily IgG autoantibodies that attach to platelets and megakaryocytes [5], specifically targeting common surface antigens, including glycoprotein (GP) αIIbβ3 (GPIIbIIIA) and GPIb-IX-V [6]. Platelets that have autoantibody bindings are then detected by phagocytes that have Fcγ-receptors (FcγRs), which leads to increased platelet phagocytosis and destruction by antibodies, mainly in the spleen [5]. Additionally, autoantibody binding to megakaryocytes can prevent them from maturing or even destroy them [7]. Also, the hormone thrombopoietin (TPO), which is a GP produced in the liver and promotes thrombopoiesis, is unable to restore normal platelet counts. ITP can be categorized as primary, also called idiopathic, and secondary, which is caused by various illnesses like viruses, medications, autoimmune diseases, infections, and cancers [1]. The reticuloendothelial system, especially the spleen, opsonizes and destroys platelets due to autoimmune-mediated platelet death in ITP, in which the antibodies are directed against surface antigens of platelets. Thus, the platelet lifespan is reduced by both increased antibody-mediated destruction and decreased platelet synthesis [8]. The majority of ITP patients are asymptomatic. Although purpura and petechiae are frequent, life-threatening bleeding is uncommon and is typically associated with platelet counts of less than 20,000 [4].

The various conditions that mimic ITP should be considered as differentials while evaluating a patient with isolated thrombocytopenia, which are drug-induced thrombocytopenia, congenital thrombocytopenia, liver cirrhosis, viral infections, leukemia, and myelodysplasias [2]. The diagnosis of ITP is a diagnosis of exclusion. The American Society of Haematology guidelines do not routinely recommend antibody testing for the diagnosis of ITP because of the test's low sensitivity and specificity, as well as the lack of an association between antibodies and clinical outcomes [6].

As per the management of ITP, a complete blood count with peripheral blood smears along with HCV and HIV tests are done for all patients. Certain patients are also eligible for additional testing, such as thyroid profiles, coagulation, immunological investigations, and bone marrow biopsies [3]. A platelet count of less than 30,000 or less than 50,000 with evidence of severe bleeding or a danger of bleeding is an indication to treat ITP [9]. The recommendation for the treatment of any potentially fatal bleeding is intravenous immunoglobulin (IVIG), glucocorticoids, and platelet transfusions [4]. Maintaining platelet counts at a level that effectively stops spontaneous bleeding is the treatment objective for ITP and not returning them to normal [10]. Splenectomy, rituximab, azathioprine, danazol, and eltrombopag (a TPO agonist) are examples of second-line therapies [10].

One of the most frequent causes of hypothyroidism is Hashimoto's thyroiditis, which affects people who are genetically susceptible to the condition. Hypothyroidism is a condition brought on by a variety of environmental causes, such as infections and other autoimmune diseases that produce auto-antibodies against the thyroid gland [11]. The condition can range from overt hypothyroidism to subclinical hypothyroidism. Hypothyroidism symptoms include constipation, dry skin, weight gain, cold intolerance, and exhaustion may be present in the patient [6]. Routine thyroid function testing is used to make the diagnosis. Increased TSH and high titers of antithyroid peroxidase (anti-TPO) and/or antithyroglobulin (anti-TG) antibodies are typically used to identify Hashimoto's illness [10]. Normal free T4 and T3 levels are accompanied by above-normal or slightly elevated TSH in subclinical hypothyroidism. Such patients are thought to benefit from levothyroxine therapy since they have a higher chance of developing overt hypothyroidism in the future due to their mildly increased TSH and strong thyroid antibodies [11]. Treatment is recommended for all patients exhibiting clinical signs of hypothyroidism, TSH levels ≥10 mIU/L, or with goiter, infertility, and strong anti-TPO antibodies, even with subclinical hypothyroidism and TSH <10 mIU/L [9,10]. Numerous case reports have detailed the connection between autoimmune thyroid disease and the ITP. With the current literature available, the impact of treating thyroid disease with the clinical outcome of ITP remains controversial, given the majority consists of case reports and retrospective investigations [12]. Thyroid issues and ITP may point to a far more serious immunological tolerance issue, making these individuals more susceptible to refractory conditions [13].

Various researchers like Ito et al. have demonstrated that treating hypothyroidism or hyperthyroidism in certain patient groups improves the clinical prognosis of ITP [14]. Treatment of concomitant thyroid disease has been documented to either improve response to ITP medication or cause auto ITP to remit [15]. It is still up for debate whether treatment is necessary for subclinical Hashimoto's thyroiditis with higher normal TSH levels. Thus, it is possible that treating her underlying subclinical hypothyroidism may have improved her clinical response to ITP treatment.

## Conclusions

Thyroid dysfunction, especially subclinical manifestations, is common in ITP patients. Sometimes it may contribute to exacerbation of symptoms of ITP. In most of the cases, it does not have a significant impact on the disease response to treatment. In a patient having concomitant ITP with auto-immune hypothyroidism, treating both conditions simultaneously has a better response than treating either one alone. However, more studies are needed to establish an association between thyroid dysfunction and ITP.

## References

[1] Kiefel V, Santoso S, Mueller-Eckhardt C (1992). Serological, biochemical, and molecular aspects of platelet autoantigens. Semin Hematol.

[2] Yelne P, Kabra R, Mathurkar S, Gaidhane SA, Acharya S, Kumar S (2022). Relationship between vaccination and immune thrombotic thrombocytopenia: coincidental or causal?. Cureus.

[3] Bowles KM, Turner GE, Wimperis JZ (2004). Resolution of chronic severe refractory thrombocytopenia after treatment of hypothyroidism. J Clin Pathol.

[4] Cheung E, Liebman HA (2009). Thyroid disease in patients with immune thrombocytopenia. Hematol Oncol Clin North Am.

[5] McMillan R (2007). The pathogenesis of chronic immune thrombocytopenic purpura. Semin Hematol.

[6] Chow L, Aslam R, Speck ER (2010). A murine model of severe immune thrombocytopenia is induced by antibody- and CD8+ T cell-mediated responses that are differentially sensitive to therapy. Blood.

[7] Zufferey A, Kapur R, Semple JW (2017). Pathogenesis and therapeutic mechanisms in  tmmune thrombocytopenia (ITP). J Clin Med.

[8] Provan D, Stasi R, Newland AC (2010). International consensus report on the investigation and management of primary immune thrombocytopenia. Blood.

[9] Neunert C, Lim W, Crowther M, Cohen A, Solberg L Jr, Crowther MA (2011). The American Society of Hematology 2011 evidence-based practice guideline for immune thrombocytopenia. Blood.

[10] Chang M, Nakagawa PA, Williams SA, Schwartz MR, Imfeld KL, Buzby JS, Nugent DJ (2003). Immune thrombocytopenic purpura (ITP) plasma and purified ITP monoclonal autoantibodies inhibit megakaryocytopoiesis in vitro. Blood.

[11] Manuja N, Kumar S, Acharya S, Daiya V, Sood A (2023). Simultaneous myasthenic crisis with thyrotoxic crisis in an adult male: an autoimmune overlap. Cureus.

[12] Lekurwale V, Acharya S, Shukla S, Kumar S (2023). Neuropsychiatric manifestations of thyroid diseases. Cureus.

[13] Talwar D, Kumar S, Garikapati A, Chaturvedi A (2020). Sub clinical disease presenting with serious clinical manifestations-blame thyroid. J Evolution Med Dent Sci.

[14] Ito S, Fujiwara SI, Murahashi R (2021). Clinical association between thyroid disease and immune thrombocytopenia. Ann Hematol.

[15] Tahir H, Sheraz F, Sagi J, Daruwalla V (2016). Immune thrombocytopenia (ITP) secondary to subclinical Hashimoto’s thyroiditis: role of levothyroxine in improving the clinical outcome of ITP. J Investig Med High Impact Case Rep.

